# SlotDeconv: spatial transcriptomics deconvolution via diversity-constrained prototype learning and spatial refinement

**DOI:** 10.1093/bioinformatics/btag424

**Published:** 2026-08-21

**Authors:** Hanzhang Fang, Cong Qi, Yuanjie Zou, Yeqing Chen, Zhi Wei

**Affiliations:** Department of Computer Science, New Jersey Institute of Technology, University Heights, Newark, New Jersey 07102, United States; Department of Computer Science, New Jersey Institute of Technology, University Heights, Newark, New Jersey 07102, United States; Department of Computer Science, New Jersey Institute of Technology, University Heights, Newark, New Jersey 07102, United States; Department of Computer Science, New Jersey Institute of Technology, University Heights, Newark, New Jersey 07102, United States; Department of Computer Science, New Jersey Institute of Technology, University Heights, Newark, New Jersey 07102, United States

## Abstract

**Motivation:**

Spatial transcriptomics (ST) measures gene expression in intact tissues. In spot-based ST assays, each spot can contain mixtures of multiple cell types. Deconvolution is particularly challenging when closely related cell subtypes share highly similar expression profiles and when spatial context is underutilized during proportion estimation.

**Results:**

We present SlotDeconv, a method for ST deconvolution consisting of a single-cell reference module and a spatial inference module. The reference module learns discriminative cell-type signatures using slot-based prototype vectors decoded into a reference matrix, trained with a negative binomial reconstruction loss and a max-margin diversity constraint that discourages similar cell-type signatures. Ablation studies confirm that both components are essential: removing the diversity constraint reduces spot-wise Pearson correlation by 41%, and replacing learned prototypes with cell-type mean expression reduces it to near zero. The spatial inference module initializes spot-level proportions via gene-weighted nonnegative least squares (NNLS), then refines them by minimizing Kullback–Leibler (KL) divergence between observed and reconstructed spot expression under a spatial neighborhood consistency regularizer. Benchmarked against CARD, RCTD, Cell2location, and Spotiphy on a 27 cell type mouse brain dataset, SlotDeconv achieves the highest spot wise Pearson correlation (approximately 0.56) and cosine similarity (0.633), outperforming competing methods in spot-wise correlation, with particularly strong gains on transcriptionally similar cortical neuronal subtypes. Biological validation on human pancreatic cancer and mouse olfactory bulb datasets further confirms spatial specificity.

**Availability:**

Source code is available at https://github.com/HannahNJIT/SlotDeconv

## 1 Introduction

Spatial transcriptomics (ST) quantifies gene expression together with spatial coordinates in tissue sections. In widely used platforms such as 10× Visium and Slide-seq, each spot typically captures transcripts from multiple cells, making cell-type deconvolution a prerequisite for downstream analyses such as tissue characterization and spatial pattern discovery (Ståhl *et al.* 2016, Gaspard-Boulinc *et al.* 2025).

Existing deconvolution methods differ primarily in how they construct cell-type references and whether they model spatial dependence across spots. Spot-independent approaches, including linear mixture models (RCTD (Cable *et al.* 2022) and Cell2location (Kleshchevnikov *et al.* 2022)), and probabilistic formulations (Stereoscope (Andersson *et al.* 2020), DestVI (Lopez *et al.* 2022)), treat each spot in isolation. Spatially aware methods such as CARD (Ma and Zhou 2022) and DeCoST (Guo *et al.* 2025) introduce neighborhood priors, while domain adaptation approaches (CellDART (Bae *et al.* 2022), LETSmix (Zhan *et al.* 2025)) address distributional discrepancies between scRNA-seq references and ST data. Most recently, Spotiphy (Yang *et al.* 2025) enables single-cell spatial whole-transcriptome profiling across entire tissue sections, substantially advancing the resolution and biological richness of spatial transcriptomics data.

Despite this progress, two challenges persist even in leading methods. First, closely related subtypes often exhibit highly similar expression profiles, leading to correlated reference matrices and unstable proportion estimates (Tian *et al.* 2023, Garmire *et al.* 2024). Most methods fix the reference matrix as cell-type averages or use marker genes without explicitly penalizing inter-type similarity. Second, spatial regularization reduces noise but can blur sharp biological boundaries, making smoothing strength a sensitive and dataset dependent choice (Ma and Zhou 2022, Liu *et al.* 2023).

SlotDeconv addresses both issues within an interpretable mixture model formulation. A single-cell reference module learns discriminative cell-type signatures using slot-based prototypes with a max-margin diversity constraint, explicitly discouraging similar signatures during training. A subsequent spatial inference module initializes proportions via gene-weighted nonnegative least squares (NNLS) and refines them with a Kullback–Leibler (KL) divergence objective and spatial neighborhood consistency regularizer. Our contributions are: (1) a reference learning strategy that improves separation of closely related subtypes through learned prototypes and diversity regularization; (2) a spatial refinement step that improves deconvolution accuracy through spatial neighborhood information; and (3) strong performance on a 27 cell type mouse brain ST benchmark, with particularly strong gains on transcriptionally similar cortical neuronal subtypes.

## 2 Materials and methods

### 2.1 Overview

**Figure 1:**
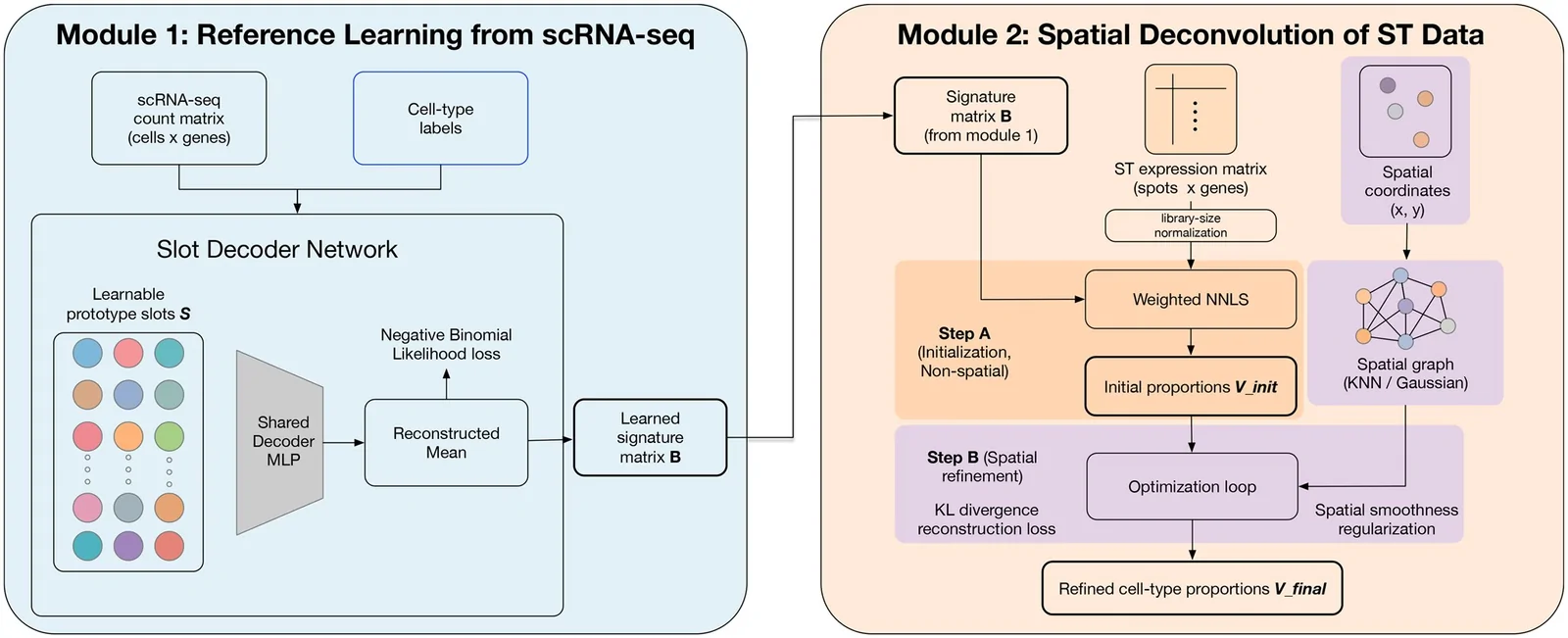
Overview of SlotDeconv. The single-cell reference module learns discriminative cell-type signatures using slot-based prototypes with a diversity constraint. The spatial inference module initializes proportions via weighted NNLS, then refines them using KL divergence reconstruction with spatial neighborhood consistency.

SlotDeconv deconvolves spatial transcriptomics data using a single-cell reference module that learns discriminative cell-type signatures and a spatial inference module that estimates spot-wise cell-type proportions (Fig. 1).

### 2.2 Data preprocessing

#### 2.2.1 Cell balancing

Let $Y\in R_{+}^{N_{c}\times G_{0}}$ denote the scRNA-seq count matrix, where $N_{c}$ is the number of cells, $G_{0}$ the number of genes, $Y_{cg}$ the observed count of gene *g* in cell *c*, and $\tau_{c}\in \{1,\ldots ,K\}$ the cell-type label of cell *c* among *K* types. To prevent abundant cell types from dominating training, we subsample each cell type to at most *M* cells (default M=750), yielding a balanced training set with $N_{c}^{\prime }$ cells.

#### 2.2.2 Gene selection

For each gene $g\in \{1,\ldots ,G_{0}\}$, we compute a discriminative score $w_{g}$ using an ANOVA-style ratio on the balanced scRNA-seq data. Let $n_{k}$ denote the number of balanced cells with label $\tau_{c}=k$. The within-type mean of gene *g* in cell type *k* is


(1) $$\mu_{kg}=\frac{1}{n_{k}}\sum_{c:\tau_{c}=k}Y_{cg}.$$

The global mean of gene *g* across all balanced cells is


(2) $$\mu_{g}=\frac{1}{N_{c}^{\prime }}\sum_{c=1}^{N_{c}^{\prime }}Y_{cg}.$$

The pooled between-type variance proxy is


(3) $${\text{Var}}_{\text{between}}(g)=\frac{1}{N_{c}^{\prime }}\sum_{k=1}^{K}n_{k}{\left(\mu_{kg}-\mu_{g}\right)}^{2}.$$

The within-type variance of gene *g* in cell type *k* is


(4) $$\sigma_{kg}^{2}=\frac{1}{n_{k}}\sum_{c:\tau_{c}=k}{\left(Y_{cg}-\mu_{kg}\right)}^{2},$$

and the pooled within-type variance proxy is


(5) $${\text{Var}}_{\text{within}}(g)=\frac{1}{N_{c}^{\prime }}\sum_{k=1}^{K}n_{k}\sigma_{kg}^{2}.$$

We define


(6) $$w_{g}=\frac{{\text{Var}}_{\text{between}}(g)}{{\text{Var}}_{\text{within}}(g)+10^{-6}}.$$

We select the top *G* genes (default G=2500) with highest $w_{g}$ and intersect them with genes present in the ST data. After selection, we re-index retained genes as g∈{1,…,G} and restrict all subsequent computations—including the count matrices Y and X—to these *G* genes. The gene weight vector $w={\left(w_{1},\ldots ,w_{G}\right)}^{⊤}$ is fixed after preprocessing and reused in the spatial inference module.

##### Library size normalization

For scRNA-seq cell *c*, define its library size on the *G* selected genes as $s_{c}=\sum_{g=1}^{G}Y_{cg}$. Let $S=\{s_{c^{\prime }}:c^{\prime }\in \{1,\ldots ,N_{c}^{\prime }\},s_{c^{\prime }}>0\}$ denote the set of strictly positive library sizes across all balanced cells. The median-normalized scRNA size factor is


(7) $${\tilde{s}}_{c}=\frac{\max(s_{c},10^{-8})}{\text{median}(S)}.$$

For ST spot *i*, let $X\in R_{+}^{N\times G}$ denote the ST count matrix on the same *G* genes, where *N* is the number of spots and $X_{ig}$ is the count of gene *g* in spot *i*. Define the spot library size as $l_{i}=\sum_{g=1}^{G}X_{ig}$, and let $L=\{l_{i^{\prime }}:i^{\prime }\in \{1,\ldots ,N\},l_{i^{\prime }}>0\}$ be the set of strictly positive spot library sizes. The median-normalized ST size factor is


(8) $${\tilde{l}}_{i}=\frac{\max(l_{i},10^{-8})}{\text{median}(L)}.$$

### 2.3 Single-cell reference module

This module learns a cell-type signature matrix $B\in R_{+}^{K\times G}$ from the balanced scRNA-seq data (recall: *K* cell types, *G* selected genes). Learnable parameters are: (i) slot prototypes ${\left\{s_{k}\right\}}_{k=1}^{K}$, (ii) decoder weights θ, and (iii) dispersion raw parameters $\alpha^{\text{raw}}\in R^{G}$. Observed counts Y, labels $\left\{\tau_{c}\right\}$, and size factors $\left\{{\tilde{s}}_{c}\right\}$ are treated as constants.

#### 2.3.1 Slot-based signature learning

For each cell type k∈{1,…,K}, we maintain a learnable prototype vector $s_{k}\in R^{d}$ with d=128. A shared decoder $f_{\theta }:R^{d}\to R^{G}$ maps each slot to gene-space logits:


(9) $$h_{k}=f_{\theta }(s_{k}).$$

We convert logits to an unnormalized nonnegative signature by


(10) $${\tilde{b}}_{kg}=\text{softplus}(\text{clip}(h_{kg},-20,20)),\;g\in \{1,\ldots ,G\},$$

where $\text{softplus}(x)=\text{log}(1+e^{x})$ and clip(x,a,b)=min(max(x,a),b). The signature matrix B is obtained by row-normalization:


(11) $$B_{kg}=\frac{{\tilde{b}}_{kg}}{\sum_{g^{\prime }=1}^{G}{\tilde{b}}_{kg^{\prime }}+10^{-8}}.$$

With the $10^{-8}$ stabilizer, each row sums approximately to one.

#### 2.3.2 Negative binomial reconstruction loss

For a scRNA-seq cell *c* with label $\tau_{c}=k$, we model each observed count $Y_{cg}$ using a Negative Binomial distribution $Y_{cg}∼\text{NB}(\mu_{cg},\alpha_{g})$, where the mean is


(12) $$\mu_{cg}={\tilde{s}}_{c}\cdot {\tilde{b}}_{kg}.$$

The gene-specific dispersion parameter is parameterized as


(13) $$\alpha_{g}=\text{softplus}(\alpha_{g}^{\text{raw}})+10^{-6},$$

where $\alpha_{g}^{\text{raw}}\in R$ is learnable. The variance is $\text{Var}(Y_{cg})=\mu_{cg}+\mu_{cg}^{2}/\alpha_{g}$. The NB log-likelihood under mean–shape parameterization is


(14) $$\begin{array}{cc} \;\text{log}\;p_{\text{NB}}(y;\mu ,\alpha )=\text{log}\;\Gamma (y+\alpha )-\text{log}\;\Gamma (\alpha )-\text{log}\;\Gamma (y+1) & \\ +\alpha \;\text{log}\;\frac{\alpha }{\alpha +\mu }+y\;\text{log}\;\frac{\mu }{\alpha +\mu }. & \end{array}$$

In implementation, μ is clamped to $\left[10^{-8},10^{8}\right]$, α is clamped to $\left[10^{-4},10^{4}\right]$, and $10^{-12}$ is added inside logarithms for numerical stability. The reconstruction loss is the average negative log-likelihood:


(15) $$L_{\text{NB}}=-\frac{1}{N_{c}^{\prime }G}\sum_{c=1}^{N_{c}^{\prime }}\sum_{g=1}^{G}\;\text{log}\;p_{\text{NB}}(Y_{cg};\mu_{cg},\alpha_{g}).$$

#### 2.3.3 Diversity loss

To encourage discriminative signatures across cell types, we penalize high cosine similarity between signature rows. Let $B_{k}={\left(B_{k1},\ldots ,B_{kG}\right)}^{⊤}$ denote the *k*-th row of B. Define the $\ell_{2}$-normalized signature by


(16) $${\hat{B}}_{k}=\frac{B_{k}}{\|B_{k}{\|}_{2}},\;\|B_{k}{\|}_{2}=\sqrt{\sum_{g=1}^{G}B_{kg}^{2}}.$$

The cosine similarity is $S_{jk}={\hat{B}}_{j}^{⊤}{\hat{B}}_{k}\in [0,1]$. The diversity loss is


(17) $$L_{\text{div}}=\underset{j\neq k}{\max}{\left[S_{jk}-m\right]}_{+},$$

where m=0.1 and ${\left[x\right]}_{+}=\max(x,0)$. The reference objective is


(18) $$L_{\text{ref}}=L_{\text{NB}}+\lambda_{\text{div}}L_{\text{div}},$$

where $\lambda_{\text{div}}=5.0$.

#### 2.3.4 Reference module optimization

We minimize $L_{\text{ref}}$ with respect to ${\left\{s_{k}\right\}}_{k=1}^{K}$, θ, and $\alpha^{\text{raw}}$ using AdamW (learning rate $10^{-3}$, weight decay $10^{-5}$) with cosine annealing over 2500 epochs; gradients are clipped to maximum norm 5.0. After training, B is computed and kept fixed for the spatial inference module.

### 2.4 Spatial inference module

Let $X\in R_{+}^{N\times G}$ denote the ST count matrix on the selected gene set, where *N* is the number of spots and $X_{ig}$ is the observed count of gene *g* in spot *i*. Let $P\in R^{N\times 2}$ denote the spatial coordinates, where $p_{i}=(p_{i1},p_{i2})$ is the (x,y) position of spot *i*. In this module, X, P, $\left\{{\tilde{l}}_{i}\right\}$, w, and B are fixed constants, and the only learnable variables are logits $L\in R^{N\times K}$ (hence V).

The goal is to estimate the proportion matrix $V\in R_{+}^{N\times K}$, where $V_{ik}$ represents the fraction of cell type *k* in spot *i*, satisfying $\sum_{k=1}^{K}V_{ik}=1$ and $V_{ik}\geq 0$.

#### 2.4.1 Initialization via weighted NNLS

For spot *i*, define the library-size normalized expression ${\tilde{x}}_{ig}=X_{ig}/{\tilde{l}}_{i}$ for each gene g∈{1,…,G}, where ${\tilde{l}}_{i}$ is the fixed size factor from preprocessing. Under a linear mixture model, we approximate ${\tilde{x}}_{ig}\approx \sum_{k=1}^{K}v_{ik}B_{kg}$. We compute transformed gene weights as


(19) $${\tilde{w}}_{g}=\text{clip}\left({\left(\frac{w_{g}}{\text{median}(w)+10^{-8}}\right)}^{\gamma },0.2,5.0\right),$$

Where γ=0.8 and median(w) denotes the median of $\left(w_{1},\ldots ,w_{G}\right)$. For each spot *i*, we solve weighted NNLS:


(20) $${\hat{v}}_{i}=\arg\underset{v_{i}\geq 0}{\min}\sum_{g=1}^{G}{\tilde{w}}_{g}{\left({\tilde{x}}_{ig}-\sum_{k=1}^{K}B_{kg}v_{ik}\right)}^{2}.$$

Let ${\hat{v}}_{ik}$ denote the *k*-th component of ${\hat{v}}_{i}$. We project onto the simplex by


(21) $$V_{ik}^{\text{init}}=\{\begin{array}{cc} {\hat{v}}_{ik}/\sum_{k^{\prime }=1}^{K}{\hat{v}}_{ik^{\prime }} & \text{if}\;\sum_{k^{\prime }=1}^{K}{\hat{v}}_{ik^{\prime }}>0,\\ 1/K & \text{otherwise}. \end{array}$$

Stacking all spots yields $V^{\text{init}}\in R_{+}^{N\times K}$.

#### 2.4.2 Spatial refinement

##### 2.4.2.1 Softmax parameterization

We introduce learnable logits $L\in R^{N\times K}$ and compute proportions by row-wise softmax:


(22) $$V_{ik}=\frac{\;\text{exp}(L_{ik})}{\sum_{k^{\prime }=1}^{K}\;\text{exp}\;(L_{ik^{\prime }})}.$$

Logits are initialized from NNLS by


(23) $$L_{ik}^{\left(0\right)}=\text{log}(V_{ik}^{\text{init}}+10^{-6}).$$

##### 2.4.2.2 KL reconstruction loss

Define the observed gene-composition distribution of spot *i* as


(24) $$q_{ig}=\frac{X_{ig}}{\sum_{g^{\prime }=1}^{G}X_{ig^{\prime }}+10^{-8}}.$$

The predicted gene composition is


(25) $${\hat{r}}_{ig}=\sum_{k=1}^{K}V_{ik}B_{kg}.$$

We normalize the prediction to mitigate numerical drift:


(26) $$r_{ig}=\frac{{\hat{r}}_{ig}}{\sum_{g^{\prime }=1}^{G}{\hat{r}}_{ig^{\prime }}+10^{-8}}.$$

The KL reconstruction loss is


(27) $$L_{\text{KL}}=\frac{1}{N}\sum_{i=1}^{N}\sum_{g=1}^{G}q_{ig}\left(\text{log}(q_{ig}+10^{-8})-\text{log}(r_{ig}+10^{-8})\right).$$

##### 2.4.2.3 Spatial affinity matrix

We construct a fixed spatial affinity matrix $A\in R_{+}^{N\times N}$ from coordinates P. Let ${\bar{p}}_{1}=\frac{1}{N}\sum_{i=1}^{N}p_{i1}$ and ${\bar{p}}_{2}=\frac{1}{N}\sum_{i=1}^{N}p_{i2}$ be the coordinate means, and let $\sigma_{p_{1}}$ and $\sigma_{p_{2}}$ be the coordinate standard deviations. The z-score normalized coordinates are


(28) $${\tilde{p}}_{i1}=\frac{p_{i1}-{\bar{p}}_{1}}{\sigma_{p_{1}}+10^{-8}},\;{\tilde{p}}_{i2}=\frac{p_{i2}-{\bar{p}}_{2}}{\sigma_{p_{2}}+10^{-8}}.$$

The pairwise distance is $d_{ij}=\sqrt{{\left({\tilde{p}}_{i1}-{\tilde{p}}_{j1}\right)}^{2}+{\left({\tilde{p}}_{i2}-{\tilde{p}}_{j2}\right)}^{2}}$. For dense graphs, we set $\sigma_{\text{rbf}}=\text{median}(\{d_{ij}:i\neq j,d_{ij}>0\})$; for sparse κ-NN graphs (default κ=15, used when N>8000), we set $\sigma_{\text{rbf}}=\text{median}(\{d_{ij}:j\in N_{\kappa }(i),d_{ij}>0\})+10^{-8}$. The unnormalized affinities are


(29) $${\tilde{A}}_{ij}=\{\begin{array}{cc} \;\text{exp}\;\left(-\frac{d_{ij}^{2}}{2\sigma_{\text{rbf}}^{2}}\right) & i\neq j,\\ 0 & i=j, \end{array}$$

and row-normalization yields $A_{ij}={\tilde{A}}_{ij}/(\sum_{j^{\prime }}{\tilde{A}}_{ij^{\prime }}+10^{-8})$.

##### 2.4.2.4 Spatial consistency loss

Let $V^{\text{nbr}}=\mathrm{AV}$ denote neighborhood-averaged proportions. The spatial consistency loss is the mean squared deviation:


(30) $$L_{\text{sp}}=\frac{1}{NK}\sum_{i=1}^{N}\sum_{k=1}^{K}{\left(V_{ik}-V_{ik}^{\text{nbr}}\right)}^{2}.$$

##### 2.4.2.5 Spatial module objective and optimization

The spatial refinement objective is


(31) $$L_{\text{spatial}}=L_{\text{KL}}+\lambda_{\text{sp}}L_{\text{sp}},$$

Where $\lambda_{\text{sp}}=10.0$. We minimize $L_{\text{spatial}}$ with respect to logits L using Adam (learning rate $10^{-2}$) with cosine annealing over 500 epochs. The final output is V=softmax(L) applied row-wise.

### 2.5 Implementation details

SlotDeconv is implemented in PyTorch. Unless otherwise noted, it uses G=2500 selected genes, at most 750 balanced cells per cell type, $\lambda_{\text{div}}=5.0$, margin m=0.1, $\lambda_{\text{sp}}=10.0$, κ=15, and gene-weight exponent γ=0.8. The decoder $f_{\theta }$ is a 3-layer MLP with hidden dimensions (256,512), LayerNorm and ReLU activations after each hidden layer, and dropout rate 0.1 after the first hidden layer. The NNLS initialization uses scipy.optimize.nnls. All hyperparameters are summarized in Table S1, available as supplementary data at *Bioinformatics* online; additional modeling choices are detailed in Supplementary Note S2, available as supplementary data at *Bioinformatics* online.

### 2.6 Evaluation metrics

We assess deconvolution accuracy using spot-wise metrics between predicted proportions V and ground-truth proportions $V^{*}$.

*Spot-wise PCC and RMSE*. For each spot *i*, we compute Pearson correlation and RMSE between predicted and ground-truth proportion vectors $v_{i}$ and $v_{i}^{*}$ across cell types, and report the mean over spots.

*Spot-wise JSD*. We compute the Jensen–Shannon distance using scipy.spatial.distance.jensenshannon with base = 2.0 on smoothed distributions and square it to obtain JSD:


(32) $${\text{JSD}}_{i}={\left(\text{JSdist}(v_{i}+\epsilon_{m},v_{i}^{*}+\epsilon_{m};\text{base}=2)\right)}^{2},\;\epsilon_{m}=10^{-12},$$

where $v_{i}$ and $v_{i}^{*}$ are the predicted and ground-truth proportion vectors for spot *i* normalized to sum to one.

*Spot-wise cosine similarity*. For each spot *i*, we compute cosine similarity between predicted and ground-truth proportion vectors:


(33) $${\text{Cosine}}_{i}=\frac{v_{i}^{⊤}v_{i}^{*}}{\|v_{i}{\|}_{2}\|v_{i}^{*}{\|}_{2}+10^{-12}}.$$

We report the mean over spots.

*AUPR*. For a given evaluation subset (27 cell types for the benchmark; 26 valid cell types for the ablation), we binarize each spot–cell-type pair by positive ground-truth proportion and compute one flattened precision–recall AUC from predicted proportions.

## 3 Results

### 3.1 Benchmarking on the mouse brain dataset

**Figure 2:**
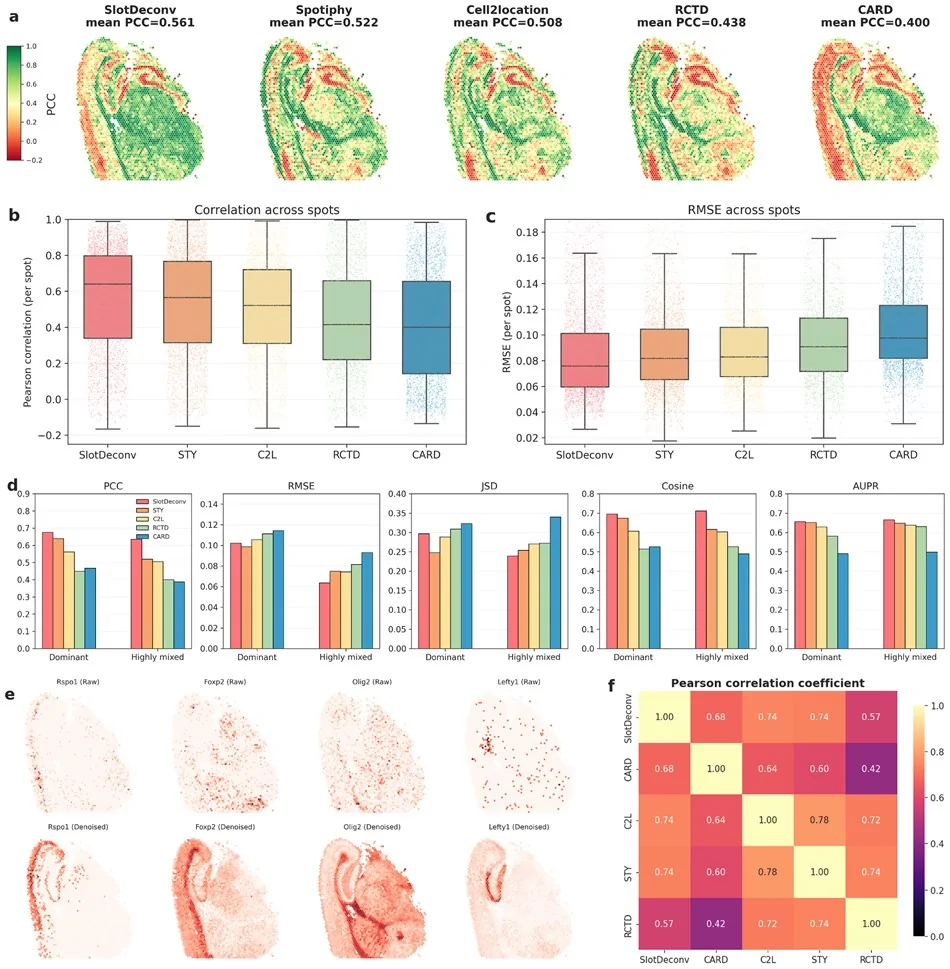
Benchmarking SlotDeconv on the Spotiphy mouse brain dataset. (a) Per spot PCC maps; red (low) to green (high). (b, c) Per spot PCC (b; higher is better) and RMSE (c; lower is better). STY, Spotiphy; C2L, Cell2location. (d) Stratified performance: *Dominant* (max proportion ∈[0.5,0.9]) vs. *Highly mixed* (<0.5). (e) Raw counts (top) vs. reconstructed profiles (bottom); $\hat{X}=V\hat{B}$. (f) Cross method concordance heatmap. Methods: SlotDeconv, Spotiphy (Yang *et al*. 2025) (STY), Cell2location (Kleshchevnikov *et al*. 2022) (C2L), RCTD (Cable *et al*. 2022), CARD (Ma and Zhou 2022). 3243 spots with matched ground truth.

We benchmarked SlotDeconv on the Spotiphy mouse brain dataset (Yang *et al.* 2025), which provides spot level ground truth cell-type proportions for 3243 Visium spots by spatially aligning matched Xenium measurements from an adjacent tissue section across 27 cell types (see Supplementary Note S1, available as supplementary data at *Bioinformatics* online for dataset construction and preprocessing details). We compared SlotDeconv against four reference based deconvolution baseline methods: Spotiphy (Yang *et al.* 2025), Cell2location (Kleshchevnikov *et al.* 2022), RCTD (Cable *et al.* 2022), and CARD (Ma and Zhou 2022). SlotDeconv achieves the highest mean spot-wise PCC (0.561) and the lowest mean RMSE (0.085) among all methods (Fig. 2b, c; Supplementary Tables S7–S8, available as supplementary data at *Bioinformatics* online). Spatial maps of spot-wise PCC further show that SlotDeconv maintains stronger and more spatially coherent agreement with ground truth across the tissue than competing methods (Fig. 2a; spots where all five methods yield negative PCC are characterized in Table S9, available as supplementary data at *Bioinformatics* online). A cross method concordance analysis shows that SlotDeconv remains broadly consistent with other methods while yielding more accurate spot level estimates (Fig. 2f). Stratified evaluation shows that SlotDeconv remains consistently superior on both dominant and highly mixed spots (Fig. 2d), indicating robust performance under challenging mixing conditions. Runtime and memory use are reported in Table S11, available as supplementary data at *Bioinformatics* online.

Reconstructed marker expression ($\hat{X}=V\hat{B}$) is markedly more spatially coherent than raw spot level signals (Fig. 2e), supported by strong global geometry preservation (Mantel r=0.727) and local neighborhood consistency (kNN overlap=0.303, k=20; Table S6, available as supplementary data at *Bioinformatics* online).

To isolate the contribution of each component, we conducted ablation experiments on valid cell types only, removing (i) the diversity loss ($\lambda_{\text{div}}=0$) and (ii) the slot-based reference module (replaced by per-type mean expression). Both components are essential: removing diversity loss reduces PCC by 41% (0.558→0.327), while replacing the slot reference with simple averaging renders deconvolution ineffective (PCC close to zero; Table 1). Notably, spatial refinement cannot compensate for poor signatures. It degrades performance without diversity loss, confirming that accurate reference learning is a prerequisite for effective spatial modeling.

**Table 1:** Ablation study on the mouse brain benchmark (valid cell types only, excluding the constant ground truth Neutrophil class).

Configuration	Stage	RMSE↓	JSD↓	PCC↑	Cosine↑	AUPR↑
**SlotDeconv (Full)**	NNLS	0.102	0.479	0.409	0.528	0.561
	+Spatial	**0.094**	**0.406**	**0.558**	**0.633**	**0.590**
**w/o Diversity loss**	NNLS	0.107	0.496	0.345	0.456	0.476
	+Spatial	0.111	0.515	0.327	0.434	0.446
**w/o Slot reference**	NNLS	0.157	0.860	− 0.068	0.066	0.250
	+Spatial	0.151	0.850	− 0.021	0.112	0.292

Each row removes one component from the full model. Bold indicates best per column.

The learned reference signatures exhibit low cross-type similarity (off-diagonal cosine: mean=0.114, max=0.136) and strong marker specificity (23.7× enrichment over random; 27/27 types significant at p<0.05; Fig. S1, available as supplementary data at *Bioinformatics* online) and show concordance with independent Xenium expression specificity for many cortical subtypes (Table S10, available as supplementary data at *Bioinformatics* online). In prototype weight space, clustering quality improves substantially over PCA embeddings (Silhouette: 0.067→0.378; ARI: 0.154→0.509).

Pathway enrichment recovers expected annotations for 24/27 cell types, including glutamate transport for astrocytes, B-cell receptor signaling for B cells, myelination for oligodendrocytes, and synapse pruning for microglia (Table S2, available as supplementary data at *Bioinformatics* online). Remaining assignment errors concentrate in transcriptionally similar excitatory subtypes that are predominantly confused with oligodendrocytes, consistent with ambient signal leakage (Fig. S3, available as supplementary data at *Bioinformatics* online).

### 3.2 SlotDeconv characterizes spatial heterogeneity in human pancreatic ductal adenocarcinoma

We next evaluated SlotDeconv on a human pancreatic ductal adenocarcinoma (PDAC) spatial transcriptomics dataset with pathologist annotated tissue regions (Cancer, Stroma, Duct epithelium, and Pancreatic parenchyma) provided by prior work (Moncada *et al.* 2020). The PDAC section contains 426 spots and 15 324 genes, and the scRNA-seq reference includes 1,926 cells annotated into 20 cell types. Because ground truth spot level cell-type proportions are unavailable in this real-tissue setting, we assessed (i) spatial consistency with annotated compartments, (ii) concordance between inferred components and marker gene expression, (iii) differential abundance between tumor and non-tumor spots, and (iv) co-occurrence patterns across inferred components.

**Figure 3:**
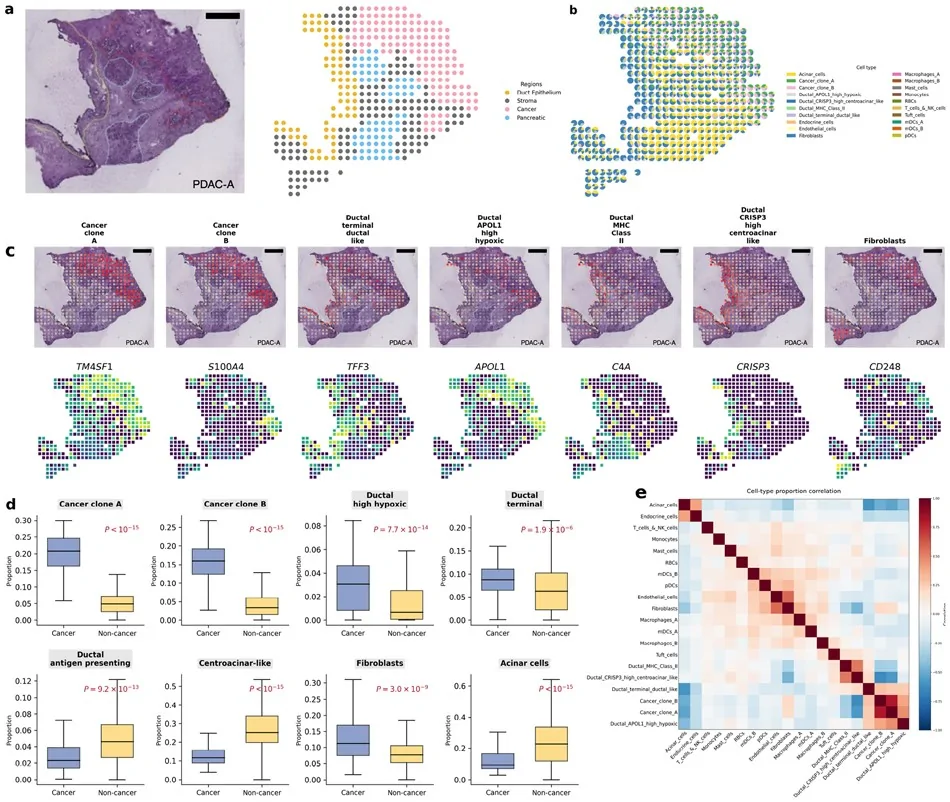
PDAC spatial analysis. (a) H&E image and spot level region annotations. (b) Pie-chart map of inferred cell-type compositions at each spot. (c) Spatial distribution of representative inferred components (top) and corresponding marker gene expression (bottom): *TM4SF1*, *S100A4*, *TFF3*, *APOL1*, *C4A*, *CRISP3*, and *CD248*. (d) Differential abundance between Cancer and Non-cancer spots (two-sided Mann–Whitney *U* test). (e) Spot wise correlation matrix of inferred cell-type proportions.

SlotDeconv recapitulated the spatial architecture of the PDAC section (Fig. 3a and b), producing coherent compartmentalization of malignant, ductal, stromal, and pancreatic parenchymal components. To quantify spatial consistency with annotated regions, we computed region specific AUC scores by treating each compartment as a one versus rest classification problem using inferred proportions as scores. Cancer clone A and Cancer clone B achieved AUC values of 0.949 and 0.931 for the Cancer region, respectively; Ductal CRISP3 high centroacinar like cells showed high specificity for Duct epithelium (AUC = 0.925); and Acinar cells for Pancreatic parenchyma (AUC = 0.863).

To validate biological fidelity, we compared inferred component maps to the spatial expression of representative marker genes (Fig. 3c). Tumor associated components aligned with tumor markers (e.g. Cancer clone A with *TM4SF1* and Cancer clone B with *S100A4*), ductal components aligned with ductal markers (e.g. Ductal terminal ductal-like with *TFF3*, Ductal APOL1 high hypoxic with *APOL1*, and CRISP3 high centroacinar like with *CRISP3*), and stromal components aligned with stromal markers (e.g. Fibroblasts with *CD248*). Spearman correlation analysis supported this concordance, with Acinar cells reaching ρ=0.871 and Cancer clone A reaching ρ=0.671 between inferred proportions and marker-gene expression (Table S3, available as supplementary data at *Bioinformatics* online).

We then performed tumor versus non-tumor differential analysis (Fig. 3d). Malignant components were strongly enriched in Cancer spots (Cancer clone A: 3.5 fold, $P<10^{-50}$; Cancer clone B: 3.4-fold, $P<10^{-46}$), while exocrine programs were depleted (CRISP3 high centroacinar-like: $P<10^{-42}$; Acinar cells: $P<10^{-16}$). The Ductal APOL1-high hypoxic component was enriched in Cancer spots ($P=7.7\times 10^{-14}$), with a substantial shift in median proportion (0.0306 in Cancer vs. 0.00694 in Non-cancer) and a rank-biserial effect size of 0.45, consistent with tumor associated hypoxia. Fibroblasts were moderately elevated in Cancer regions ($P=3.0\times 10^{-9}$), reflecting cancer associated stromal remodeling. Notably, the antigen-presenting ductal component (MHC class II) was higher in Non-cancer spots ($P=9.2\times 10^{-13}$), consistent with its association with non-tumor ductal epithelium. Because the Non-cancer group aggregates multiple annotated compartments (Stroma, Duct epithelium, and Pancreatic parenchyma), we additionally report tumor-versus-non-tumor comparisons in Table S5, available as supplementary data at *Bioinformatics* online.

Finally, spot wise correlation analysis revealed structured co-occurrence patterns across inferred components (Fig. 3e). The Ductal APOL1-high hypoxic state co-varied with malignant components (Spearman ρ=0.529 with Cancer clone A and ρ=0.408 with Cancer clone B), whereas CRISP3 high centroacinar-like cells were anti-correlated with malignant signals (Spearman ρ=−0.548 with Cancer clone A and ρ=−0.616 with Cancer clone B), indicating spatial segregation between exocrine and malignant programs. Collectively, these results demonstrate that SlotDeconv yields spatially coherent and biologically interpretable deconvolution on complex PDAC tissue.

### 3.3 Validation on the Mouse Olfactory Bulb

We next evaluated SlotDeconv on the Mouse Olfactory Bulb (MOB) spatial transcriptomics dataset, which exhibits well-characterized laminar organization across four anatomical layers: olfactory nerve layer (ONL), glomerular layer (GL), mitral cell layer (MCL), and granule cell layer (GCL) (Cable *et al.* 2022, Kleshchevnikov *et al.* 2022). The scRNA-seq reference contains 12 801 cells across five cell types: granule cells (GC), periglomerular cells (PGC), olfactory sensory neurons (OSNs), mitral/tufted cells (M_TC), and external plexiform layer interneurons (EPL_IN). A total of 277 spots with manual layer annotations were used for evaluation.

**Figure 4:**
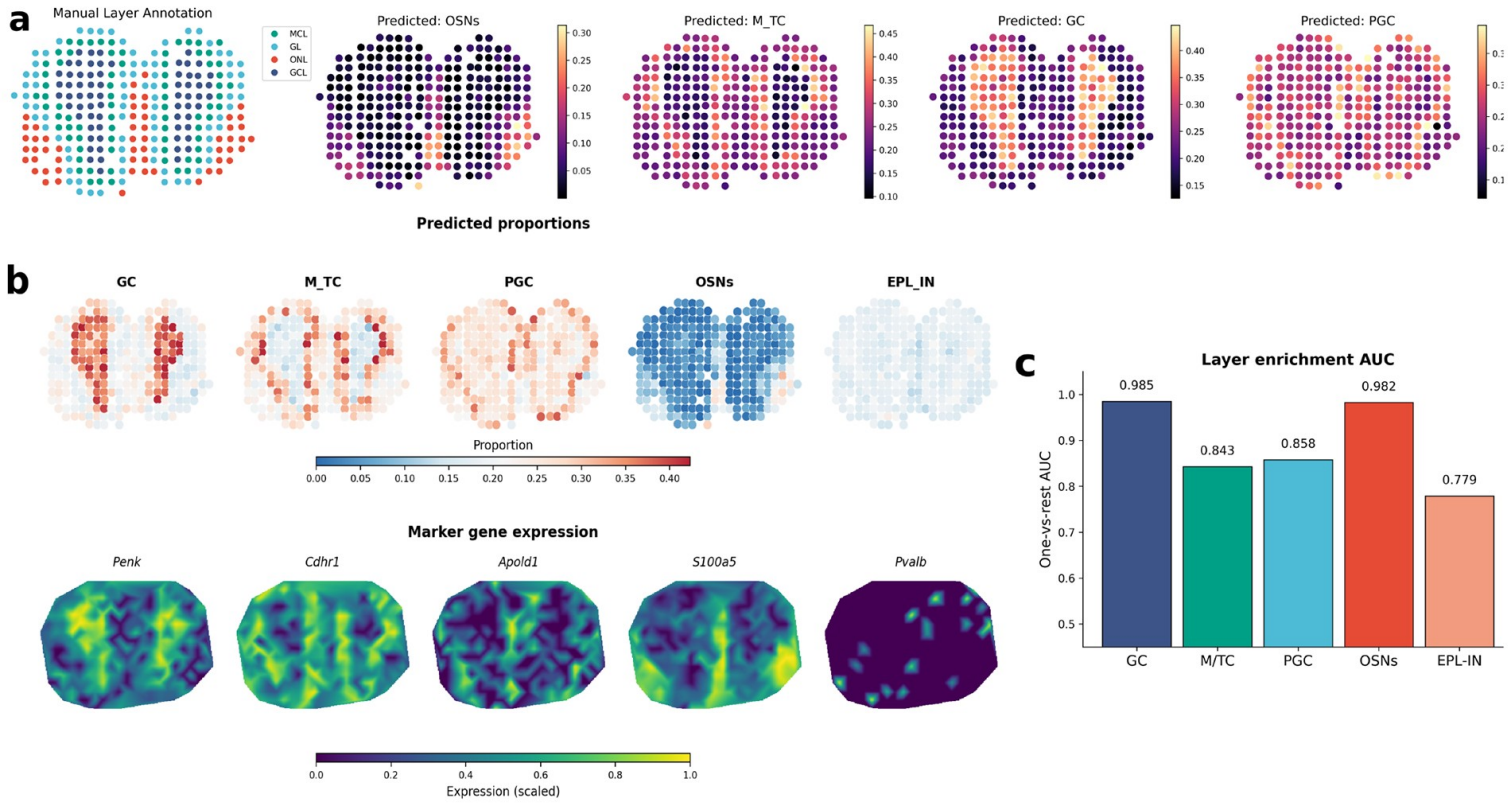
SlotDeconv validation on the Mouse Olfactory Bulb (MOB) dataset. (a) Manual layer annotation (left) and SlotDeconv-predicted proportions for four representative cell types (right). Layer colors: ONL (red), GL (blue), MCL (green), GCL (dark blue). (b) Predicted proportions (top) and corresponding marker gene expression (bottom) for each cell type. Markers: *Penk* (GC), *Cdhr1* (M_TC), *Apold1* (PGC), *S100a5* (OSNs), *Pvalb* (EPL_IN). (c) Per-cell-type layer enrichment AUC with best-matching layer annotated. 277 spots, 5 cell types, 4 anatomical layers.

SlotDeconv recovered laminar organization consistent with MOB anatomy without using layer labels during training or inference (Fig. 4a). Layer enrichment analysis confirmed strong spatial specificity: GC→GCL (AUC=0.985), OSNs→ONL (AUC=0.982), PGC→GL (AUC=0.858), M_TC→MCL (AUC=0.843), and EPL_IN→MCL (AUC=0.779); all associations are significant (Kruskal–Wallis $p<10^{-14}$; Fig. 4c). The weaker EPL_IN localization is consistent with limited reference support (161 cells, 1.3% of reference) and shared GABAergic signatures among interneuron populations. Predicted proportions correlate with canonical marker gene expression (Spearman ρ: OSNs=0.699, M_TC=0.639, GC=0.518, PGC=0.407; Fig. 4b), confirming that inferred programs capture genuine spatial signals.

Dominant-type assignment achieved ARI=0.349 and Purity=0.646 with respect to manual layer labels (Fig. S4a, available as supplementary data at *Bioinformatics* online), comparable to CARD (ARI=0.356, Purity=0.675) and outperforming Cell2location (0.326, 0.625), RCTD (0.190, 0.520), and Spotiphy (0.056, 0.419). The inferred proportion correlation structure reveals expected co-variation among inner-layer inhibitory populations and anti-correlation between OSNs and inner-layer cell types (Fig. S4b, available as supplementary data at *Bioinformatics* online).

## 4 Discussion and conclusions

SlotDeconv separates spatial deconvolution into signature learning and proportion inference. This decoupled design makes each component’s contribution explicit: ablation confirms that both the slot based reference module and spatial refinement are essential, with removing either causing substantial or catastrophic performance loss (Table 1). The modularity also improves interpretability, as reconstructed marker patterns are smooth and biologically coherent without confounding spatial priors with biological programs. Across tissues without spot level ground truth, SlotDeconv yields consistent results: inferred compartments agree with pathologist annotations in PDAC and recover expected laminar organization in MOB.

Limitations include sensitivity to reference quality and granularity, potential oversmoothing at sharp tissue boundaries, and the current focus on single section inference without explicit batch correction. Closely related populations remain partially confounded when reference profiles are highly similar. Future directions include uncertainty aware reference learning, boundary adaptive spatial regularization, and multi sample extensions.

## Supplementary Material

btag424_Supplementary_Data

## Data Availability

Source code, scripts, and accession links needed to reproduce the analyses are available at https://github.com/HannahNJIT/SlotDeconv. Public datasets used in this study are described in Supplementary Note S1, available as supplementary data at *Bioinformatics* online.
