# Supplementary material for: SlotDeconv: spatial transcriptomics deconvolution via diversity-constrained prototype learning and spatial refinement
Source: Bioinformatics. 2026 Aug 21;42(Suppl 2):btag424. doi: 10.1093/bioinformatics/btag424 (PMC13501309; doi:10.1093/bioinformatics/btag424)
Supplement: Supplementary file 1 [file btag424_supplementary_data.pdf]

Supplementary Materials

Supplementary Note S1: Datasets

Mouse brain dataset. The mouse brain benchmark was obtained from the Spotiphy study (Yang et al., 2025, Zenodo record 10520022). Ground-truth cell-type proportions were derived from Xenium-based high-resolution spatial profiling of an adjacent tissue section from the same specimen, with spot-level proportions defined by spatial alignment of Xenium measurements to Visium spots (Spotiphy Supplementary Table S3). This matched experimental design provides experimentally grounded proportion estimates rather than simulated mixtures. The benchmark comprises 3,243 Visium spots with matched ground truth (a subset of the full 3,476-spot grid restricted to regions with aligned Xenium measurements), 32,285 genes, and 24,627 reference single cells across 27 cell types. The scRNA-seq reference was constructed by integrating non-immune populations from the Allen Brain Map mouse whole cortex and hippocampus atlas with $CD45^+$ immune cells from the Spotiphy immune-enriched component, supplemented by Spotiphy mouse brain pool cells for five cell types not directly mappable from Allen metadata (CA, SUB, Microglia, L4_IT_CTX, L5_6_IT_CTX). One cell type (Neutrophil) has constant zero ground-truth proportions across all spots; the ablation analysis in the main text excludes this constant class and uses the remaining 26 valid cell types.

PDAC dataset. The human PDAC dataset (PDAC-A section) was obtained from Moncada et al. (2020) (GEO: GSE111672), comprising 426 spatial spots and matched H&E imaging with expert region annotations (Cancer, Stroma, Duct epithelium, Pancreatic parenchyma). The scRNA-seq reference contains 1,926 cells across 20 annotated cell types. Ground-truth spot-level proportions are unavailable in this real-tissue setting; validation was performed using spatial concordance with annotated compartments and marker gene expression concordance.

Mouse Olfactory Bulb dataset. The MOB dataset was obtained from the Spatial Transcriptomics Research website, comprising 277 spots with manual layer annotations across four anatomical layers (ONL, GL, MCL, GCL). The matched scRNA-seq reference (GEO: GSE121891) contains 12,801 cells across five cell types: granule cells (GC), periglomerular cells (PGC), olfactory sensory neurons (OSNs), mitral/tufted cells (M_TC), and external plexiform layer interneurons (EPL_IN). Layer annotations were used only for post-hoc evaluation and were not provided to the model during training or inference.

Supplementary Note S2: Modeling choices

The gene score w_g is large when between-type variation exceeds within-type variation, so it prioritizes genes that help separate cell types. The softplus transformation enforces nonnegative reference signatures, while clipping logits before softplus improves numerical stability. The max-margin diversity loss penalizes the most similar pair of signatures at each update, acting as a hard-negative constraint without further separating pairs already below the margin.

Supplementary Figures and Tables

Table S1: **Default SlotDeconv hyperparameters used in the main analyses.** Values are those specified in the Methods section.

Component	Hyperparameter	Value
Gene selection	Selected genes G	2500
Cell balancing	Maximum cells per type M	750
Reference module	Prototype dimension d	128
Reference module	Decoder hidden dimensions	256, 512
Reference module	Dropout rate	0.1
Reference optimization	Optimizer	AdamW
Reference optimization	Learning rate	10^{-3}
Reference optimization	Weight decay	10^{-5}
Reference optimization	Training epochs	2500
Reference optimization	Gradient clipping norm	5.0
Diversity constraint	Weight λ_{div}	5.0
Diversity constraint	Margin m	0.1
NNLS initialization	Gene-weight exponent γ	0.8
NNLS initialization	Gene-weight clipping range	0.2–5.0
Spatial graph	Neighbors κ	15
Spatial refinement	Weight λ_{sp}	10.0
Spatial refinement	Optimizer	Adam
Spatial refinement	Learning rate	10^{-2}
Spatial refinement	Refinement epochs	500

Table S2: **Pathway enrichment of program-specific markers.** For each representative cell type, enrichment analysis (GO Biological Process 2021 and KEGG 2021) is performed on the top 100 program-specific genes. The most significant term passing adjusted $p < 0.05$ is shown. Full results for all 27 cell types are available in the accompanying data files; 24/27 types have at least one significant pathway.

Cell type	Database	Term	Adj. p	Representative genes
Astro	GO BP	neurotransmitter uptake	3.58×10^{-3}	SLC1A3, ATP1A2, GLUL
BCell	GO BP	B cell receptor signaling	2.80×10^{-7}	CD79A, CD79B, CD19, SYK
Oligo	GO BP	myelination	5.40×10^{-5}	PLP1, MBP, SOX10
Endo	GO BP	blood vessel morphogenesis	1.76×10^{-7}	KDR, ENG, SOX17, SOX18
Microglia	GO BP	synapse pruning	1.54×10^{-9}	C1QA, C1QB, TREM2, CX3CR1
Pvalb	GO BP	potassium ion transport	1.27×10^{-4}	KCNC1, KCNC2, KCNAB1
Sst	GO BP	glutamatergic synaptic transm.	1.08×10^{-3}	GRIK5, GRIK3, GRIK1
TCell	GO BP	T cell receptor signaling	1.16×10^{-8}	LCK, CD3E, CD3G, PTPRC

Table S3: **PDAC marker concordance between inferred proportions and marker-gene expression.** For each inferred cell-type component, we compute Spearman correlation between the predicted proportions and the mean log1p-CPM expression of automatically selected marker genes from the scRNA-seq reference (top entries shown).

Cell type	n_{markers}	ρ	p-value	Markers
Acinar_cells	5	0.871	3.57×10^{-133}	CTRB2, CTRB1, PRSS1, CPB1, PRSS2
Ductal_CRISP3_high_centroacinar_like	5	0.712	4.65×10^{-67}	REG3A, CRISP3, REG1A, MMP1, MUC5B
Cancer_clone_A	5	0.671	3.98×10^{-57}	TM4SF1, CLDN1, KRT17, S100P, S100A14
Cancer_clone_B	5	0.635	2.11×10^{-49}	KRT17, S100A4, KRT16, SLC20A1, S100P
Fibroblasts	5	0.588	5.68×10^{-41}	COL1A1, COL3A1, COL1A2, IGFBP5, LUM
Mast_cells	5	0.528	5.98×10^{-32}	TPSB2, TPSAB1, CPA3, RGS1, SRGN
Ductal_APOL1_high_hypoxic	5	0.456	2.88×10^{-23}	APOL1, DUOX2, CLDN4, TACSTD2, SOX4
mDCs_B	5	0.439	1.73×10^{-21}	SRGN, APOE, C1QA, LAPTM5, C1QB

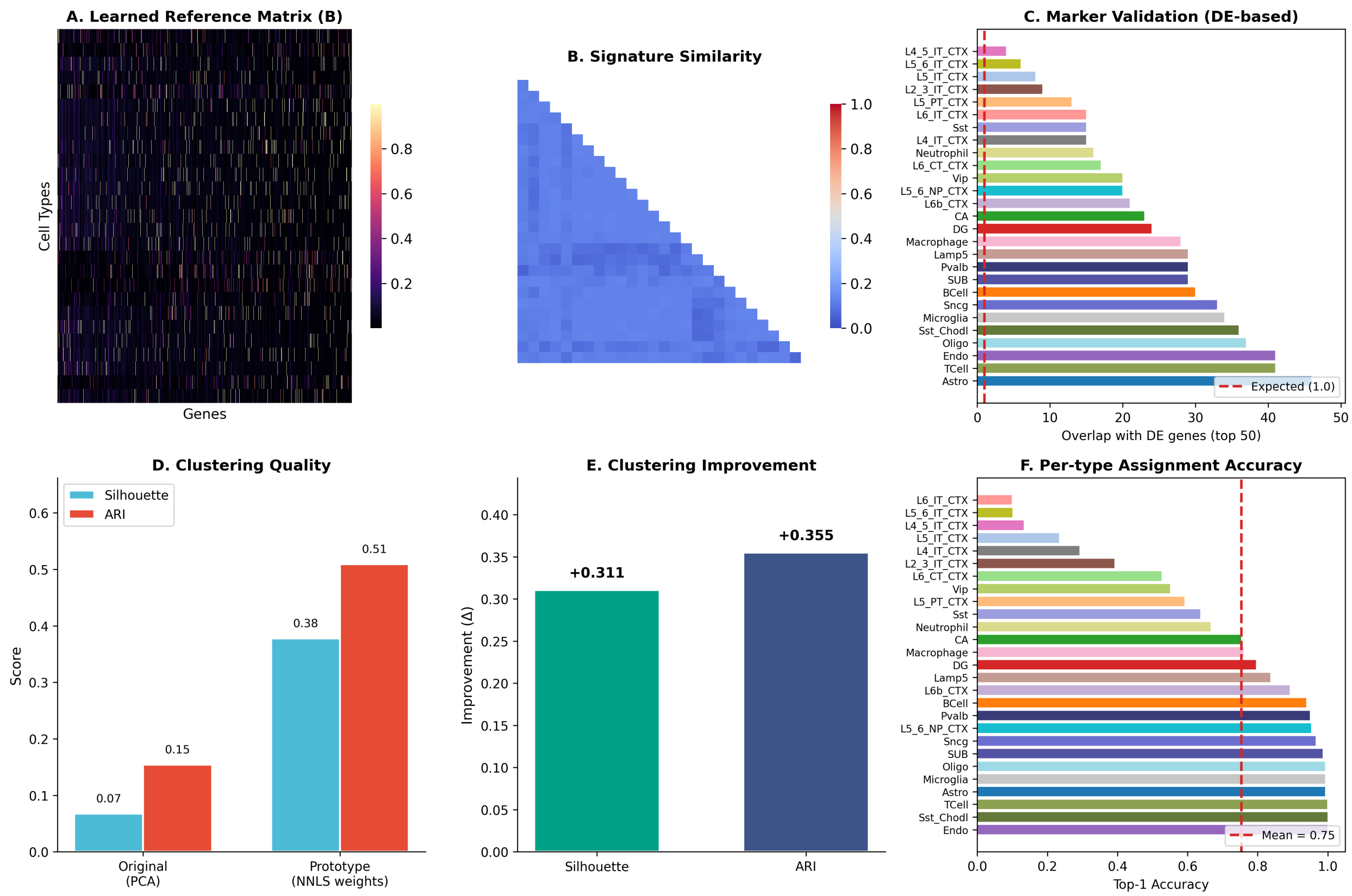

Figure S1: **Unified validation of learned reference programs and prototypes on the mouse brain dataset.** **(a)** Learned reference matrix $\hat{\mathbf{B}}$ (cell types $\times$ genes), illustrating sparse and cell-type-specific expression programs. **(b)** Pairwise cosine similarity between learned programs (off-diagonal mean $= 0.114$, max $= 0.136$), confirming low cross-type redundancy. **(c)** Self-consistent marker validation: overlap between program-specific top genes and independently identified DE genes (top 50 per type). Average overlap is 23.7 genes with 23.7$\times$ enrichment over random expectation; all 27 cell types are significant ($p < 0.05$). **(d)** Clustering quality in conventional PCA embedding versus prototype-weight space, reporting Silhouette score (0.067$\to$0.378) and ARI (0.154$\to$0.509). **(e)** Absolute improvement in clustering metrics (ΔSilhouette$=$ +0.311; ΔARI$=$ +0.355). **(f)** Per-type top-1 assignment accuracy based on prototype weights (mean $= 0.754$; range 0.099–1.000). Dashed line indicates mean accuracy.

Table S4: **PDAC region specificity quantified by one-versus-rest AUC.** For each inferred cell-type component, we compute the best region and the corresponding AUC (top entries shown).

Cell type	Best region	AUC
Cancer_clone_A	Cancer	0.949
Cancer_clone_B	Cancer	0.931
Ductal_CRISP3_high_centroacinar_like	Duct Epithelium	0.925
Acinar_cells	Pancreatic	0.863
Ductal_MHC_Class_II	Duct Epithelium	0.826
Ductal_terminal_ductal_like	Duct Epithelium	0.734
Ductal_APOL1_high_hypoxic	Cancer	0.724
mDCs_B	Duct Epithelium	0.707

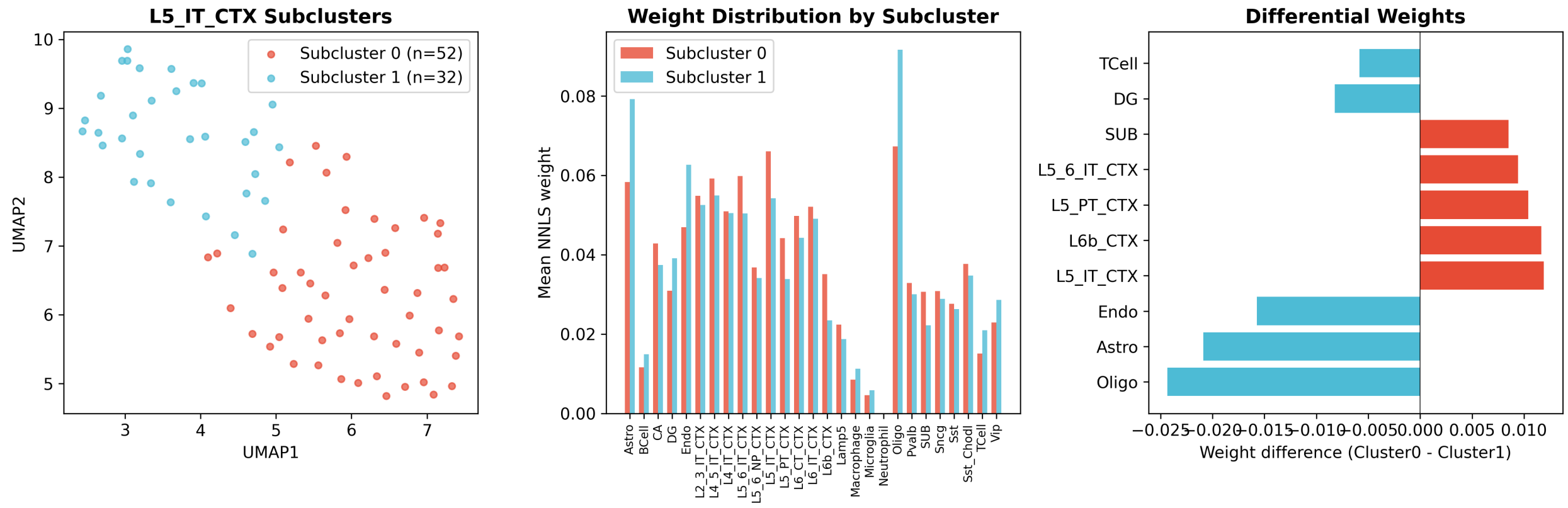

Figure S2: **Within-type heterogeneity analysis for L5_IT_CTX in prototype-weight space. (a)** UMAP visualization of L5_IT_CTX cells colored by k-means subcluster assignment ($k = 2$). **(b)** Mean NNLS prototype-weight distribution across all reference programs for each subcluster. **(c)** Differential prototype weights (subcluster 0 minus subcluster 1) for programs with the largest shifts. The most differentially expressed marker between subclusters is *Plp1* ($p = 0.0008$), an oligodendrocyte marker, consistent with variable ambient or myelin-associated signal contamination.

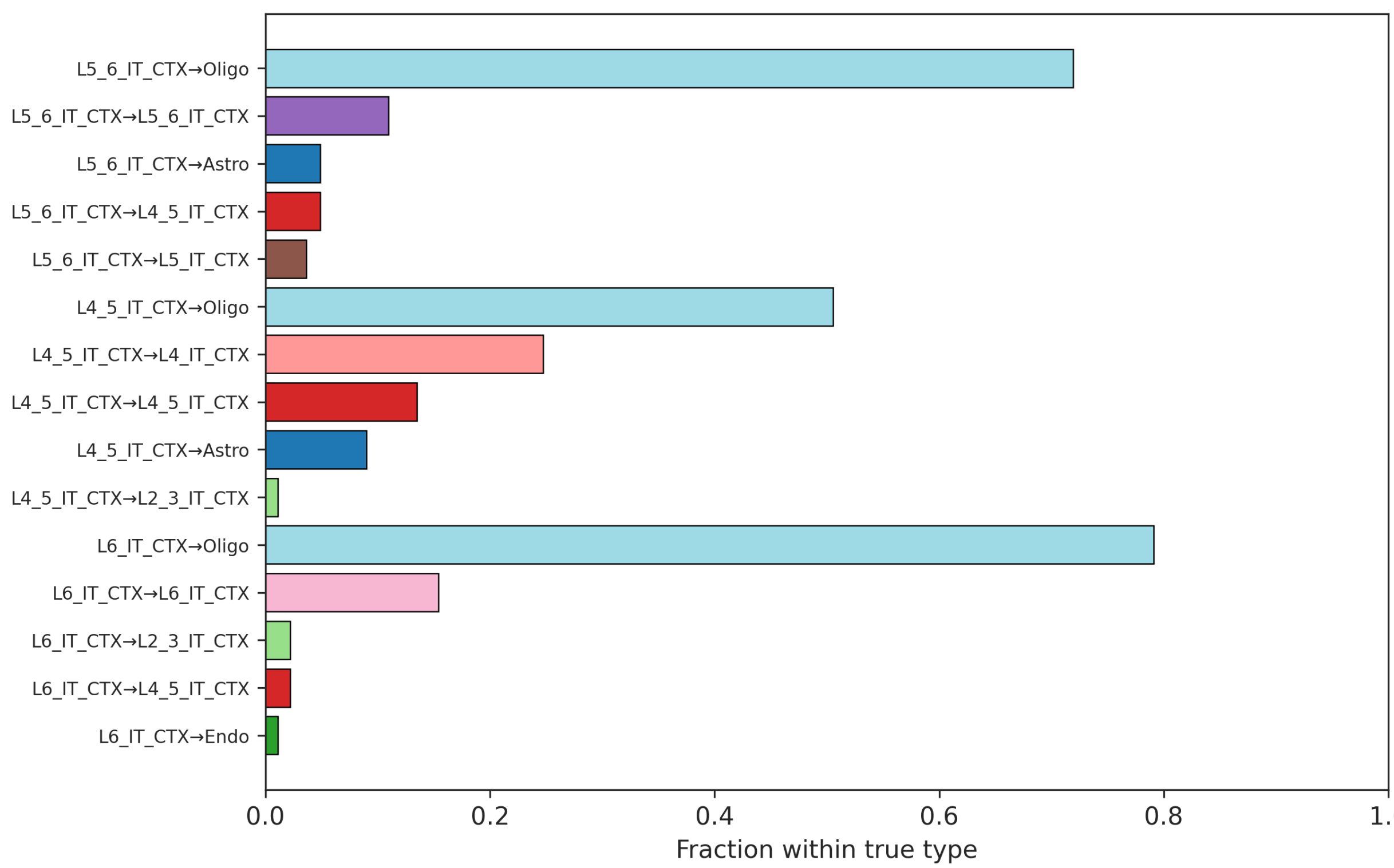

Figure S3: **Error characterization for the three lowest-accuracy excitatory subtypes.** For L5_6_IT_CTX, L4_5_IT_CTX, and L6_IT_CTX, the fraction of cells assigned to the top predicted types is shown. These types are predominantly misassigned as Oligo, consistent with transcriptional overlap and ambient signal leakage in myelinated cortical regions.

Table S5: **PDAC differential abundance between Cancer and Non-cancer spots (two-sided Mann–Whitney U test).** We report mean proportions in Cancer vs Non-cancer spots, fold change (Cancer/Non-cancer), and p-values (top entries shown).

Cell type	Mean(Cancer)	Mean(Non-cancer)	Fold change	p-value
Cancer_clone_A	0.199	0.056	3.545	9.37×10^{-51}
Cancer_clone_B	0.156	0.045	3.445	6.94×10^{-47}
Ductal_CRISP3_high_centroacinar_like	0.127	0.270	0.471	1.73×10^{-43}
Acinar_cells	0.129	0.259	0.500	4.08×10^{-17}
Ductal_APOL1_high_hypoxic	0.030	0.015	1.965	7.69×10^{-14}
Ductal_MHC_Class_II	0.027	0.048	0.563	9.15×10^{-13}
Fibroblasts	0.127	0.092	1.388	3.04×10^{-9}
mDCs_B	0.005	0.012	0.456	9.03×10^{-7}

Table S6: **Quantitative validation of marker-gene reconstruction structure.** We report global structure preservation between raw and reconstructed expression profiles using Mantel correlation, and local neighborhood consistency in expression space using kNN overlap ($k = 20$).

Metric	Value
Mantel correlation (r)	0.7271
kNN overlap ($k = 20$)	0.3028

Table S7: **Mouse brain benchmark summary across all compared methods.** Values are mean benchmark metrics on the 27-cell-type mouse brain evaluation used for Figure 2. PCC and Cosine are mean spot-wise metrics; AUPR is computed as a single value over flattened spot–cell-type pairs. The ablation analysis in Table 1 uses the 26 valid cell types after excluding the constant-zero Neutrophil class and is therefore not directly comparable to this benchmark across all metrics. All reported predictions were evaluated on the same 3,243 spots and 27 cell-type columns; the CARD row uses the full-reference no-down-sampling run.

Method	PCC↑	RMSE↓	Cosine↑	AUPR↑	JSD↓
SlotDeconv	**0.561**	**0.085**	**0.633**	**0.590**	0.406
Spotiphy	0.522	0.088	0.598	0.538	**0.396**
Cell2location	0.508	0.089	0.590	0.561	0.416
RCTD	0.438	0.094	0.532	0.519	0.423
CARD	0.400	0.104	0.486	0.425	0.497

Table S8: **Bootstrap uncertainty and paired spot-level tests on the mouse brain benchmark.** Top: metric means and 95% bootstrap confidence intervals from 1,000 spot-level resamples ($n = 3{,}243$). Bottom: paired two-sided Wilcoxon signed-rank p-values comparing SlotDeconv against each baseline on the same spots. All paired tests favor SlotDeconv except JSD versus Spotiphy, where Spotiphy has the lower (better) JSD. AUPR is reported in Table S7 but omitted from the spot-level bootstrap intervals and paired tests because it is computed as one flattened ranking metric per method rather than one paired value per spot.

Method	PCC↑	RMSE↓	Cosine↑	JSD↓
SlotDeconv	0.561 [0.552, 0.571]	0.085 [0.084, 0.086]	0.633 [0.625, 0.641]	0.406 [0.401, 0.411]
Spotiphy	0.522 [0.513, 0.531]	0.088 [0.087, 0.089]	0.598 [0.591, 0.607]	0.396 [0.390, 0.401]
Cell2location	0.508 [0.499, 0.518]	0.089 [0.088, 0.090]	0.590 [0.582, 0.597]	0.416 [0.411, 0.421]
RCTD	0.438 [0.428, 0.448]	0.094 [0.093, 0.096]	0.532 [0.524, 0.541]	0.423 [0.416, 0.428]
CARD	0.400 [0.390, 0.410]	0.104 [0.103, 0.105]	0.486 [0.478, 0.495]	0.497 [0.492, 0.503]
Paired Wilcoxon p-value, SlotDeconv vs. listed baseline				
Spotiphy	1.0×10^{-55}	1.1×10^{-68}	7.4×10^{-67}	8.5×10^{-3}
Cell2location	8.5×10^{-68}	1.2×10^{-77}	3.0×10^{-75}	2.0×10^{-27}
RCTD	2.4×10^{-109}	7.2×10^{-129}	1.5×10^{-119}	4.1×10^{-38}
CARD	$< 10^{-300}$	$< 10^{-300}$	$< 10^{-300}$	$< 10^{-300}$

Table S9: **Per-method negative-PCC spot count and characterization of spots where all methods fail.** Top: number of mouse-brain spots whose spot-wise Pearson correlation against the matched-Xenium ground truth is negative for each method ($n = 3{,}243$ spots total). Bottom: comparison between the 67 spots where *all five methods* yield negative PCC ("all-methods-negative") versus the remaining 3,176 spots; Mann–Whitney U test for continuous features and Fisher's exact for the binary dominant-correct rate. All-methods-negative spots are enriched for highly dominant ground-truth compositions (median max proportion 0.887 vs. 0.444, mostly CA, DG, and L4_IT_CTX); for SlotDeconv, these spots have the true dominant type receiving near-zero mass and ranked far worse (median rank 22 vs. 2) than on typical spots. Top contributing true-dominant cell types: CA (31), DG (24), L4_IT_CTX (7).

Method	Negative-PCC spots	% of 3,243
SlotDeconv	110	3.4%
Spotiphy	151	4.7%
Cell2location	152	4.7%
RCTD	225	6.9%
CARD	320	9.9%
**All methods negative**	**67**	**2.1%**

Feature (median)	All-neg ($n = 67$)	Other ($n = 3{,}176$)	p-value
Max true proportion	0.887	0.444	2.7×10^{-32}
True entropy (nats)	0.520	1.458	6.2×10^{-29}
SlotDeconv pred. at true dominant	∼0	0.178	7.1×10^{-39}
SlotDeconv true-dominant rank	22	2	4.7×10^{-42}
SlotDeconv per-spot PCC	−0.081	0.652	2.5×10^{-44}
SlotDeconv dominant-correct rate	0%	41.3%	$8.9 \times 10^{-16\dagger}$

†Fisher's exact test; all other p-values are Mann–Whitney U.

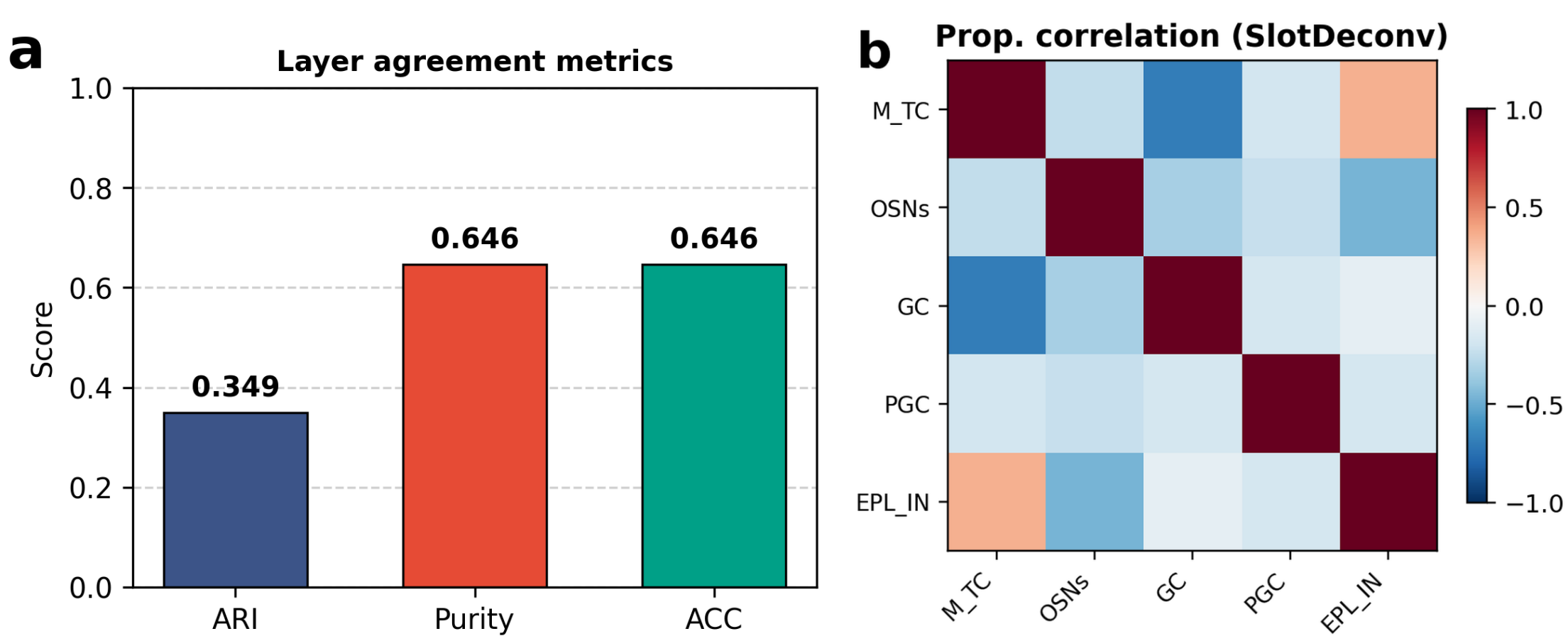

Figure S4: **Additional validation on the Mouse Olfactory Bulb (MOB) dataset.** **(a)** Layer agreement metrics (ARI, Purity, ACC) between dominant cell-type assignments and manual layer annotations. **(b)** Pearson correlation between inferred cell-type proportions, showing expected co-variation among inner-layer inhibitory populations (GC, PGC, EPL_IN) and anti-correlation between OSNs and inner-layer types.

Table S10: **Xenium shared-gene marker concordance for SlotDeconv reference signatures.** For each of 26 mouse-brain cell types, we compute Spearman correlation between SlotDeconv per-type specificity (log-ratio of $\hat{\mathbf{B}}$ vs. the mean of other cell types) and Xenium logFC (in-type mean expression vs. out-of-type mean expression) over the 159 genes shared between the Xenium panel and SlotDeconv's selected genes. This is a gene-level marker-concordance analysis, not a pathway-level enrichment comparison; the Xenium panel is itself enriched for cortical neuronal markers, so many cortical excitatory and inhibitory subtypes show strong concordance (top of table), whereas concordance is limited for non-neuronal and immune populations (microglia, oligo, astro, B/T cells, macrophage). "Top-10 overlap" counts genes in the top-10 of both rankings; hypergeometric p-values are computed against the 159-gene shared pool.

Cell type	Spearman ρ	p-value	Top-10 overlap	Hypergeom. p
L5_6_IT_CTX	0.753	2.4×10^{-30}	7	3.1×10^{-8}
L5_IT_CTX	0.745	2.0×10^{-29}	3	1.7×10^{-2}
L5_PT_CTX	0.727	1.9×10^{-27}	6	2.0×10^{-6}
L6_IT_CTX	0.716	2.6×10^{-26}	6	2.0×10^{-6}
L6_CT_CTX	0.701	8.4×10^{-25}	4	1.4×10^{-3}
L2_3_IT_CTX	0.678	1.0×10^{-22}	6	2.0×10^{-6}
SUB	0.635	2.6×10^{-19}	5	7.0×10^{-5}
Sst_Chodl	0.630	5.9×10^{-19}	6	2.0×10^{-6}
L5_6_NP_CTX	0.609	1.7×10^{-17}	7	3.1×10^{-8}
Pvalb	0.608	1.9×10^{-17}	6	2.0×10^{-6}
Lamp5	0.608	2.0×10^{-17}	8	2.3×10^{-10}
L4_5_IT_CTX	0.598	8.5×10^{-17}	6	2.0×10^{-6}
Sncg	0.588	3.7×10^{-16}	4	1.4×10^{-3}
L6b_CTX	0.558	2.0×10^{-14}	6	2.0×10^{-6}
CA	0.554	3.4×10^{-14}	4	1.4×10^{-3}
L4_IT_CTX	0.541	1.7×10^{-13}	5	7.0×10^{-5}
Vip	0.504	1.2×10^{-11}	4	1.4×10^{-3}
Sst	0.480	1.6×10^{-10}	4	1.4×10^{-3}
DG	0.467	5.3×10^{-10}	6	2.0×10^{-6}
Endo	0.133	0.095	6	2.0×10^{-6}
Microglia	−0.029	0.72	5	7.0×10^{-5}
Macrophage	−0.043	0.59	6	2.0×10^{-6}
Oligo	−0.060	0.45	7	3.1×10^{-8}
Astro	−0.069	0.39	8	2.3×10^{-10}
BCell	−0.102	0.20	2	0.12
TCell	−0.104	0.19	3	1.7×10^{-2}

Table S11: **SlotDeconv runtime on the mouse brain benchmark.** Runtime was measured on the 3,243-spot mouse brain benchmark with 24,627 reference cells, 32,285 input genes, and 27 cell types. Runs used one NVIDIA A100 GPU node (21 CPU cores and 80 GB host RAM requested). Model-stage times are mean ± s.d. over three repeated runs. CSV loading and gene alignment were measured once; the end-to-end total includes this I/O and alignment step. Peak GPU memory is the PyTorch peak allocated memory; peak host memory is the Slurm MaxRSS value.

Stage / resource	Time or memory	Notes
Data loading + gene alignment	270.9 s	measured once
Reference learning	77.4 ± 2.3 s	slot reference module
NNLS initialization	8.7 ± 0.1 s	non-spatial initialization
Spatial refinement	9.9 ± 0.1 s	spatial optimization
**Model computation subtotal**	$\mathbf{96.0 \pm 2.4}$ s	reference + NNLS + spatial
**End-to-end total**	$\mathbf{366.8 \pm 2.4}$ s	including loading + alignment
Peak GPU memory	3.31 GiB	PyTorch allocated memory
Peak host memory	36.4 GiB	Slurm MaxRSS
